# Stability of and Change in Psychopathological Risk Levels in Pre-Early Adolescents before, during, and after Their Study Sojourns: A Descriptive Study

**DOI:** 10.3390/ejihpe14030042

**Published:** 2024-03-08

**Authors:** Luca Cerniglia, Silvia Cimino

**Affiliations:** 1 Faculty of Psychology, International Telematic University Uninettuno, 00186 Roma, Italy; 2 Department of Dynamic, Clinical and Health Psychology, Sapienza University of Rome, 00185 Roma, Italy; silvia.cimino@uniroma1.it

**Keywords:** psychopathological risk, study sojourn, adolescents, longitudinal study

## Abstract

Background: Adolescents can benefit from studying abroad in terms of personal development, cross-cultural encounters, and academic enrichment. This article digs into the psychological challenges that students may face before and during their study abroad, focusing on the emotional components of their experiences. Methods: The current descriptive study sought to assess the stability or change in levels of psychopathological risk in a sample of N = 195 adolescents before and during a study abroad sojourn. To examine psychological symptoms in pre-adolescents, teachers were asked to complete a report-form questionnaire. Results: The findings of this study revealed that students’ psychological functioning changed significantly after their study abroad experience. Anxious/depressed and withdrawn ratings specifically increased from the pre-sojourn assessment (T1) to the evaluation during the stay (T2), then remained consistent (T3). In addition, the scores for rule-breaking and aggressive conduct changed, increasing from T1 to T2 and then decreasing from T2 to T3. This trend implies that teenagers may struggle with behavioral challenges early in their study abroad experience, but subsequently adjust and improve their conduct as they grow more used to the new environment. Conclusions: this research offers insight into the emotional and behavioral issues that adolescents face when studying abroad, underlining the significance of treating psychopathological risk factors in this demographic.

## 1. Introduction

The prospect of studying abroad offers adolescents an opportunity for personal growth, cross-cultural experiences, and academic enrichment, and it is a practice that schools and parents are increasingly recommending and embracing [1]. However, embarking on such a journey also brings forth an array of challenges that can significantly influence the lives of these young individuals. This is particularly true as sojourns abroad are chosen by younger and younger students. While in the past travelling abroad to study was mostly prerogative of individuals in their middle adolescence, today, several families decide to offer this experience to very young students, who have been defined as pre-early adolescents [2]. These authors acknowledge that it could be debatable whether a 9–10-year-old child can be classified as an adolescent. However, research suggests that children as young as 9 to 10 years old may be regarded as pre- or early adolescents since they have traits associated with later adolescence (impulsivity, experience seeking, propensity to rely on peers rather than family as a reference group, etc.)

While students’ difficulties in adjusting to new cultures and habits, as well as their possible problems in reaching academic goals, have been largely addressed by the previous literature, possible pre-early adolescents’ emotional/behavioral problems and psychopathological symptoms have been scarcely considered [3,4]. However, these issues are equally important and should not be overlooked. It is crucial to recognize that the emotional and behavioral well-being of students is closely tied to their overall adjustment process. Neglecting to address these aspects may hinder their ability to thrive in their new environment and could potentially lead to long-term negative outcomes. Therefore, research is needed that places more emphasis on understanding and addressing the emotional and behavioral challenges faced by students in the context of cultural adjustment [5,6,7]. This article delves into the psychological difficulties adolescents may encounter before and during their study abroad sojourns, shedding light on the emotional, aspects of their experiences.

Adolescents studying abroad often find themselves in unfamiliar cultural contexts. The process of cultural adjustment, also known as culture shock, can be particularly challenging. This phase involves adapting to new customs, values, and communication styles [8,9,10]. Adolescents might experience feelings of disorientation, frustration, and homesickness as they navigate these cultural differences [11]. The absence of familiar surroundings and support networks can intensify the struggle to establish a sense of belonging in a foreign environment. Language barriers pose a significant hurdle for adolescents studying abroad. Struggling to effectively communicate with locals and fellow students can lead to feelings of isolation and frustration. In academic settings, language difficulties can hinder academic performance and impede engagement in classroom discussions. Overcoming language barriers requires time, effort, and dedication to language learning, which can be a daunting prospect for young learners [12,13].

Moreover, building meaningful relationships in a foreign environment can be challenging due to cultural differences and social norms. Adolescents may find it difficult to connect with local peers, leading to feelings of loneliness and isolation [14,15,16]. The absence of close friends and family support systems can exacerbate these feelings, potentially impacting mental well-being and academic motivation [17].

Adapting to different educational systems and teaching styles can be another source of stress. Adolescents might face challenges in adjusting to new grading systems, coursework expectations, and academic demands [18,19,20]. Navigating unfamiliar academic environments may lead to academic underperformance and a sense of inadequacy.

Despite these challenges, many adolescents develop coping strategies and resilience during their study abroad experiences. Engaging in cross-cultural activities, seeking social support, and participating in local communities can help mitigate feelings of isolation [21,22]. Additionally, utilizing available resources such as counseling services and language support can contribute to a smoother transition.

In sum, adolescents undertaking study abroad sojourns embark on a transformative journey that is not without its challenges. Cultural adjustment, language barriers, social integration, and academic differences are some of the difficulties they may encounter; the sojourn itself and the separation from the family could constitute a distressing factor for some adolescents [23,24,25]. In fact, adolescents who already have psychopathological risk factors may experience heightened levels of distress when separated from their family. This separation can disrupt their sense of security and stability, potentially exacerbating their existing mental health issues [26]. Additionally, the absence of familial support during this crucial developmental stage may hinder their ability to cope with stressors and navigate challenges effectively. Without the support and guidance of their family, these vulnerable adolescents may struggle to develop healthy coping mechanisms and seek out appropriate resources for help. This can significantly impact their long-term well-being and increase their susceptibility to developing more severe mental health disorders [27].

With proper preparation, an open mindset, and access to support systems, adolescents can navigate these challenges and emerge from their study abroad experiences with enhanced resilience, cross-cultural skills, and a broader worldview. Although included in some of the programs for adolescents’ study travel, most adolescents do not undergo any evaluation of their psychological well-being before they leave home [28,29]. It can be argued that students attending schools are part of the general population, which is classically considered as a non-refereed (non-clinical population). Therefore, it is assumed that no relevant psychopathological risk is present and no screening of possible symptoms is needed. However, especially after the COVID-19 pandemic, the level of psychopathological symptoms in the general population has greatly increased, particularly among young students. This is particularly true in countries like Italy where the rate of infection and fatalities was very high [30]. One example of this increase in psychopathological symptoms among adolescents is the rise in rates of anxiety disorders such as generalized anxiety disorder and social anxiety disorder [31,32]. Many young people are experiencing excessive worry and fear, leading to difficulties in daily functioning and social interactions. Additionally, the prevalence of depression among students has also seen a notable rise, with many experiencing persistent feelings of sadness and a loss of interest in activities they once enjoyed. For these reasons, it seems that the general population can no longer be considered as composed by non-at-risk individuals who can embark in potentially distressing experiences (e.g., abroad sojourns) without any concern.

Bearing these considerations in mind, the present descriptive study aimed to evaluate the stability of or change in the levels of psychopathological risk in a group of adolescents before and during an abroad study sojourn.

## 2. Materials and Methods

### 2.1. Participants and Procedure

This longitudinal study was conducted with three serial assessments. The initial assessment (T1) was administered before the study sojourn period, specifically six months before it (in September); the second assessment (T2) during the stay (one month into the stay (in April); the sojourn lasted two months), and the third assessment (T3) after the subjects returned home (one month after returning home; in June). The sample of this study was recruited through a sampling thanks to the collaboration of randomly selected Italian schools (selected by a computerized random selection from a list of schools that had given their consent to participate in the study). The schools informed the families about the possibility of their offspring participating in a research project to evaluate the stability of and change in psychopathological risk before, during, and after study travel. Participants were recruited according to the following inclusion criteria: (a) no referred psychiatric diagnosis; (b) no medical condition present in the subjects at the time of recruitment; (c) no current medical and/or psychological treatment. From T1 to T2, the attrition rate’s participation was 5% (students and/or families retracted the consent to the participation in the study), while no subject dropped the study from T2 to T3. These missing data were excluded from the final study’s sample.

By following this procedure, a total sample of N = 195 pre-early adolescents were included in the present study. Most of the individuals (93%) belonged to middle socio-economic status (SES). They were 52.3% females and 47.7% males, and their mean age at the time was 9.6 (SD = 0.31) years. Subjects’ parents or guardians provided a written informed consent for all the procedures. Teachers of pre-early adolescents were asked to complete the teacher report form (TRF) questionnaire to assess the psychological symptoms of each participant in their own class in each of the three waves. They were teachers of schools in the native country of the subjects, and they kept in regular and frequent contact with students when they were abroad. They also met them online in videoconferences. Following the Declaration of Helsinki, the Ethical Committee accepted the study before its start (Protocol Number: 0000989). All adolescents exceeding the clinical cut-offs for the TRF have been excluded from the study at T1 and a more detailed psychological evaluation was proposed and (if needed) an intervention coordinated with their families.

### 2.2. Tools

#### The Teacher Report Form (TRF)

The teacher report form (TRF) [33] was used to measure the social adjustment of participants. The TRF consists of 113 problem items on a Likert-type scale ranging from 0 (not true) to 2 (very true or often true). The TRF scoring profile provides a total scale score (total problems), two broad-band scale scores (internalizing and externalizing), and eight syndrome subscale scores (withdrawn, somatic complaints, anxious/depressed, social problems, thought problems, attention problems, rule-breaking behavior, and aggressive behavior). The broad-band internalizing scale score is based on the sum of the withdrawn, somatic complaints, and anxious/depressed scale scores. The broad-band externalizing scale score is based on the rule-breaking behavior and aggressive behavior scale scores. The social problems, thought problems, and attention problems syndrome subscale scores are not included on either the broad-band internalizing or externalizing scale scores. In this study, the Cronbach’s alpha for the entire scale was 0.82.

### 2.3. Statistical Analyses

As a preliminary step, descriptive analyses were performed to ascertain the variables’ normality. Results showed that our variables were normally distributed. Additionally, a post hoc power analysis was conducted using G*Power software (3.1.9.6 version) [28] to determine whether the current sample size of 195 was sufficient to detect a hypothesized incremental effect size of 0.02 (a small effect) and an alpha level of α = 0.05. Results indicated the current sample size was sufficient to achieve a power of 0.98, with a critical F value of 3.00. Lastly, to identify relevant confounder variables to include in the subsequent analyses, Multivariate ANOVA was run for exploring the impact of students’ sex on dependent variables, that is, on each TRF dimensions’ mean scores T1, T2 and T3. Results did not detect any significant impact; therefore, sex was not included in the subsequent analyses. Then, two independent series of repeated measure analyses of variance for exploring the impact of time, as a within-subject factor, on the dependent variables, that is, on the mean scores of each TRF dimension were performed. Also, growth curve model (GCM) analysis was used to describe trajectories of these dependent variables as a function of time. With three repeated measures (i.e., T1, T2, and T3), GCM were limited to linear and quadratic models: (1) a linear model with a random intercept and random slopes, which reflects linear change over time; (2) a quadratic model with a random intercept and random slopes, which reflects change that takes on a “U” or inverted “U” shape. These unconditional models —where only the intercept, linear slope, and curved slope were specified to determine the trajectories—were centered at the month during which the first data were collected (i.e., at T1) and, therefore, represented initial scores of students’ TRF scores. Model fit was evaluated using the—2 log likelihood difference tests (—2LL). All statistical analyses were carried out using the IBM SPSS statistical package version 25.0.

## 3. Results

### Stability and Change of Pre-Early Adolescents’ TRF Dimensions

Results showed that, with the exception of attention problems, students’ psychological dimensions significantly change over time (Table 1). For clarity, for each TRF subscale, we will first present the results obtained with repeated measures ANOVA and then those obtained with GCM.

Particularly, anxious/depressed and withdrawn scores (respectively: F (1195) = 6.15, *p* < 0.05, η^2^ = 0.54; F (1195) = 153.32, *p* < 0.05, η^2^ = 0.52) significantly increase from T1 to T2 (Bonferroni post hoc test *p* < 0.0001 and *p* < 0.25, respectively) and remain stable from T2 to T3 (Bonferroni post hoc test *p* < 0.0001); the—2 log likelihood model comparison tests indicated that this pattern of change over time (χ^2^(1) = 1.32, *p* > 0.05) was best described by a significant positive linear change over time, indicating a linear and increasing trajectory (F (195.000) = 109.31, *p* < 0.001).

Somatic complaints, social problems, and thought problems scores (F (1195) = 13.56, *p* < 0.001, η^2^ = 0.22) tended to significantly increase from T1 to T2 and from T2 to T3 (Bonferroni post hoc test *p* < 0.0001 and *p* < 0.005, respectively); according to the—2 log likelihood model comparison tests, this trajectory over time (χ^2^(1) = 26.72, *p* < 0.01) was best described as a linear pattern, characterized by a significant positive linear slope (F (195.001) = 28.16, *p* < 0.001). Rule breaking and aggressive behavior scores (F (1195) = 3.71, *p* < 0.05, η^2^ = 0.15) tended to significantly increase from T1 to T2 (Bonferroni post hoc test *p* < 0.0001) and to decrease from T2 to T3 (Bonferroni post hoc test *p* < 0.005); the—2 log likelihood model comparison tests suggested that, on average, their means scores over time (χ^2^(1) = 36.11, *p* < 0.01) were best described by a significant positive linear slope and a negative quadratic (curved) slope, indicating a reversed U-shaped pattern (F (195.000) = 23.13, *p* < 0.001).

## 4. Discussion

The present study aimed to investigate the stability and change of psychopathological risk levels in pre-early adolescents before, during, and after their study sojourns.

The results of this study showed that adolescents experienced significant changes in their psychological dimensions during the study abroad period. Specifically, anxious/depressed and withdrawn scores increased from the pre-sojourn assessment (T1) to the assessment during the stay (T2), remaining stable thereafter. These findings align with previous research on cultural adjustment, which suggests that adolescents often face increased emotional challenges when adapting to a new cultural context [34,35,36,37,38]. Conversely, Abu-Rayya (2013, [39]) found that immigrant adolescents did not seem maladaptive, contrary to the acculturative stress hypothesis. Other authors (e.g., [40,41,42]) suggest that differences in emotional reactions to stress, particularly within an interpersonal context, contribute to the development of differences in the manifestation of anxiety and depression during adolescence. It has also been posited that emotional problems are positively associated with adolescent adjustment in less supportive familial environments [43,44,45]. Overall, the literature suggests that the emotional challenges faced by adolescents when adapting to a new cultural context may depend on various individual, familial, and cultural contexts [46]. However, this study does not address possible predictive, moderator and/or mediator factors contributing to the stability of and change in psychopathology levels in the recruited subjects. It just aimed to convey a descriptive picture of how adolescents’ psychological well-being can vary during their study sojourn abroad. Of course, notwithstanding the longitudinal nature of the study, the limitedness of factors taken into consideration limits drawing any causal conclusion. However, this study further shows that the increase in the psychopathology levels is not temporary and limited to the period of time students are abroad. As for the symptoms of depression, withdrawal, somatic complaints, social problems, and thought problems, the intensification of psychopathological risk lasts even after the adolescents have come back home. These findings suggest that the impact of studying abroad on psychopathology levels extends beyond the duration of the study program. That implies that additional support and interventions may be necessary to address the prolonged psychological effects experienced by returning students. To the best of our knowledge, this is the first (although preliminary) study to focus on this issue.

Differently, rule-breaking and aggressive behavior scores showed an interesting pattern of change, with an increase from T1 to T2 and a subsequent decrease from T2 to T3. This pattern suggests that adolescents may initially struggle with behavioral issues during the early stages of their study abroad experience but then adapt and improve their behavior as they become more accustomed to the new environment [47,48,49]. The reversed U-shaped trajectory observed in this dimension underscores the dynamic nature of psychopathological symptoms in adolescents during study abroad sojourns.

Despite its contributions, this study has limitations that warrant consideration. First, the sample was recruited through sampling from Italian schools, which may limit the generalizability of the findings to other cultural contexts. The generalizability of results is also limited by the fact that the theoretical background used to organize the study is based on university students, or college students of similar seniority. The generalizability of these academic papers to a study of children is not addressed in the current paper, and could be limited. Further, as the assessment of the students’ symptoms has been performed by the teacher, there might be potential biases or underreporting of certain issues, especially internalizing problems that might not manifest overtly in a classroom setting. Additionally, the study relied on teacher reports to assess psychological dimensions, which may not capture the full range of adolescent experiences. Third, no causal effect between variables was explored. Future research should incorporate self-report measures and more diverse samples to enhance the robustness of the findings and more complex hypotheses should be verified in order to better comprehend the mechanisms underpinning the changes in the levels of psychopathological symptoms.

## 5. Conclusions

In conclusion, this study sheds light on the emotional and behavioral challenges faced by adolescents during study abroad sojourns, emphasizing the importance of addressing psychopathological risk factors in this population [50,51,52,53]. By understanding these challenges and implementing appropriate support systems, it could be possible to help adolescents not only to thrive academically but also to develop the resilience and well-being necessary for their personal growth and cross-cultural experiences. Moreover, these findings have several implications for educators, parents, and policymakers involved in adolescents’ study abroad programs. First, it is crucial to recognize that adolescents may experience emotional and behavioral challenges during their study abroad, and these difficulties should not be underestimated. Adequate support systems, both from schools and families, should be in place to help adolescents navigate these challenges effectively [54].

Furthermore, given the increase in psychopathological symptoms observed in this study, it is essential to consider implementing pre-departure psychological evaluations for adolescents participating in study abroad programs. Screening for psychopathological risk factors and providing appropriate interventions or support can help prevent the exacerbation of mental health issues during the sojourn [55,56,57,58,59].

Future research should explore the specific factors that contribute to the changes in psychopathological risk observed in this study. Investigating the role of cultural factors, social support, and coping mechanisms in mitigating or exacerbating mental health challenges in adolescents studying abroad can provide valuable insights for program development and support strategies.

## Figures and Tables

**Table 1:** Means (standard deviation) of TRF subscales by time of assessment.

	Time of Assessment			F
	T1	T2	T3	Time of Assessment
TRF Subscales				
**A/D**	0.49	0.65	0.61	
	±0.49	±0.52	±0.58	6.15 *
**W/D**	0.46	0.59	0.58	
	±0.72	±0.74	±0.54	153.32 ***
**SC**	0.45	0.58	0.63	
	±0.51	±0.64	±0.62	13.56 *
**SP**	0.42	0.55	0.67	
	±0.54	±0.55	±0.56	16.71 ***
**TP**	0.50	0.61	0.64	
	±0.61	±0.61	±0.42	117.21 ***
**AP**	0.50	0.50	0.51	
	±0.74	±0.74	±0.57	0.05
**R-BB**	0.46	0.54	0.44	
	±0.44	±0.61	±0.33	14.13 *
**AB**	0.42	0.53	0.41	
	±0.63	±0.57	±0.58	13.21 *
**INT**	0.46	0.57	0.69	
	±0.65	±0.67	±0.68	101.12 ***
**EXT**	0.43	0.57	0.41	
	±0.65	±0.67	±0.68	12.31 ***
**TOT**	0.47	0.56	0.62	
	±0.52	±0.61	±0.56	98.15 ***

Note. TRF = teachers report form. A/D = anxious/depressed; W/D = withdrawn/depressed; SC = somatic complaints; SP = social problems; TP = thought problems; AP = attention problems; R-BB = rule-breaking behavior; AB = aggressive behavior; INT = internalizing problems; EXT = externalizing problems; TOT = total problems. * *p* < 0.05, *** *p* < 0.001.

## Data Availability

Data will be available via request to the authors.
